# Long-Range Regulatory Polymorphisms Affecting a GABA Receptor Constitute a Quantitative Trait Locus (QTL) for Social Behavior in *Caenorhabditis elegans*

**DOI:** 10.1371/journal.pgen.1003157

**Published:** 2012-12-20

**Authors:** Andres Bendesky, Jason Pitts, Matthew V. Rockman, William C. Chen, Man-Wah Tan, Leonid Kruglyak, Cornelia I. Bargmann

**Affiliations:** 1 Howard Hughes Medical Institute, Lulu and Anthony Wang Laboratory of Neural Circuits and Behavior, The Rockefeller University, New York, New York, United States of America; 2 Department of Biology and Center for Genomics and Systems Biology, New York University, New York, New York, United States of America; 3 Department of Genetics, Stanford University School of Medicine, Stanford, California, United States of America; 4 Howard Hughes Medical Institute, Lewis-Sigler Institute for Integrative Genomics and Department of Ecology and Evolutionary Biology, Carl Icahn Laboratory, Princeton University, Princeton, New Jersey, United States of America; Yale University, United States of America

## Abstract

Aggregation is a social behavior that varies between and within species, providing a model to study the genetic basis of behavioral diversity. In the nematode *Caenorhabditis elegans*, aggregation is regulated by environmental context and by two neuromodulatory pathways, one dependent on the neuropeptide receptor NPR-1 and one dependent on the TGF-β family protein DAF-7. To gain further insight into the genetic regulation of aggregation, we characterize natural variation underlying behavioral differences between two wild-type *C. elegans* strains, N2 and CB4856. Using quantitative genetic techniques, including a survey of chromosome substitution strains and QTL analysis of recombinant inbred lines, we identify three new QTLs affecting aggregation in addition to the two known N2 mutations in *npr-1* and *glb-5*. Fine-mapping with near-isogenic lines localized one QTL, accounting for 5%–8% of the behavioral variance between N2 and CB4856, 3′ to the transcript of the GABA neurotransmitter receptor gene *exp-1*. Quantitative complementation tests demonstrated that this QTL affects *exp-1*, identifying *exp-1* and GABA signaling as new regulators of aggregation. *exp-1* interacts genetically with the *daf-7* TGF-β pathway, which integrates food availability and population density, and *exp-1* mutations affect the level of *daf-7* expression. Our results add to growing evidence that genetic variation affecting neurotransmitter receptor genes is a source of natural behavioral variation.

## Author Summary

In both animals and humans, normal individuals can behave differently in the same environment. Natural variation in behavior is partly due to genetic differences between individuals and partly due to experience. Mapping studies have demonstrated that the genetic component of natural behavioral variation is complex, with many genes that each contribute a small amount to the observed behavior. This complexity has made it difficult to identify the causative genes for individual differences. Here we use the nematode worm *C. elegans* to dissect a social behavioral trait, the propensity to aggregate with other animals in the presence of food. We find that the behavioral differences between two wild-type worm strains result from at least five genetic differences between the strains, two of which were previously known. One of the three new loci affects a receptor for the neurotransmitter GABA, which regulates excitability in the brain. In the context of previous work, we suggest that a significant number of genes that generate behavioral variation encode neurotransmitter receptors. This analysis in a model animal may help guide discoveries of the genetic variants that affect common human behavioral traits by suggesting classes of genes to examine closely.

## Introduction

Most animal and human behaviors are variable, in part due to genetic variation between individuals. Genetic mapping of strain differences in anxiety, learning, activity levels, and the response to addictive drugs in mice, as well as aggression, locomotor activity, and sensory behaviors in *Drosophila*, has demonstrated a complex genetic basis for these traits [1]–[3]. Typically, multiple loci contribute to each behavior, the contribution of each locus is small, and the effect of many individual loci depends on the genotype at other loci and on environmental conditions [1]. This genetic complexity poses challenges for the discovery of specific genetic variants that modulate behavior, and consequently only a few genes contributing to natural behavioral diversity have been definitively identified. Defining quantitative behavioral genes at a molecular level has the potential to point to classes of genes that generate behavioral variation and to provide new insights into the neuronal control of behavior.

Social behaviors are central to the survival and reproductive success of humans and animals, and defects in social cognition and interaction are core features of human autism and schizophrenia [4], [5]. Social behaviors are also variable within and between animal species, providing a starting point for genetic analysis. Animals within a species display different social behaviors based on their sex, developmental stage, reproductive status and environmental conditions [6]. In addition, these behaviors are shaped by individual genetic variation that interacts with environmental factors. For example, genetic variation among *Drosophila* males affects their territorial aggressive behavior, and this genetic variation interacts with an environmental regulator of aggression, population density [7]–[9].

Aggregation between members of a species is a social interaction that can provide direct benefits, such as the conservation of body heat [10], as well as facilitating more complex behaviors such as reproduction, migration, defense, or communal foraging [11]. However, aggregation increases competition for local resources and facilitates disease transmission, so it is not always favorable [11], [12]. Accordingly, many animals aggregate under certain conditions but not others. Aggregation behavior and its regulation by environmental and genetic factors have been studied extensively in the nematode *Caenorhabditis elegans*. *C. elegans* aggregates spontaneously on food at high population density [13], a behavior that is enhanced at high oxygen levels [14], when food is depleted [15], or under other stressful conditions. Similar behaviors are observed in other nematode species at high density [16]. The role of aggregation in *C. elegans* biology is poorly understood, but it might promote mating [17], lower oxygen to a preferred intermediate level [14], or expose young animals to pheromones that drive entry into the stress-resistant dauer larva stage [17].

Different wild-type *C. elegans* strains vary in their propensity to aggregate. The “solitary” laboratory strain N2 aggregates infrequently on a lawn of bacterial food, whereas wild-caught “social” strains aggregate at much higher rates [13], [18]. In addition to aggregating, wild social animals move quickly on food, have a stronger preference for certain pheromones, and accumulate on the oxygen-poor border of a bacterial lawn; the latter behavior is called bordering [14], [18], [19]. Aggregation and bordering typically occur under the same conditions, in part because oxygen regulates both behaviors [13]–[15], [18]–[23]. The extreme solitary behavior of the N2 strain arose as an adaptation to laboratory conditions [24], and results from a gain-of-function point mutation in the neuropeptide receptor gene *npr-1* [18] together with a loss-of-function rearrangement of the sensory globin gene *glb-5* [24], [25]. Both NPR-1 and GLB-5 act in a circuit that regulates sensitivity to aggregation-promoting environmental signals. GLB-5 acts in sensory neurons to regulate sensitivity to environmental oxygen [24], [25], whereas NPR-1 acts in an interneuron hub of a circuit that integrates aggregation-promoting sensory cues from oxygen, noxious chemicals, and pheromones [19]. Thus the genetic and environmental regulation of aggregation converge on a shared neuronal circuit to regulate behavior.

A second genetic pathway that regulates aggregation is controlled by the TGF-β homolog DAF-7, which serves a key neuroendocrine role in integrating nutrient availability, overpopulation stress, and physiology in *C. elegans* [20]. The presence of food induces *daf-7* expression in the ASI sensory neurons, whereas population density pheromones suppress *daf-7* in ASI [26], [27]. DAF-7 protein is secreted from ASI and activates the TGF-β receptors DAF-1 and DAF-4 in a variety of neurons to regulate dauer larva development, aggregation, fat accumulation, gene expression, and lifespan [20], [21], [26]–[30]. Loss-of-function mutations in *daf-7* or its receptors result in aggregation and bordering in the N2 genetic background, and aggregation is enhanced in *daf-7; npr-1* double mutants, suggesting that these two pathways act at least partly independently of one another [20], [21].

By characterizing intercrosses between the N2 laboratory strain and the Hawaiian strain CB4856, we observed variability in aggregation and bordering behavior that were not explained by the laboratory-induced *npr-1* and *glb-5* mutations. This variability suggested the existence of additional quantitative trait loci for these behaviors. Here we use a set of recombinant inbred advanced intercross lines (RIAILs) and chromosome substitution strains to probe the genetic architecture and molecular basis of natural variation in aggregation and bordering behavior between the two *C. elegans* strains, structuring the analysis to control for the strong effect of *npr-1*. We show that aggregation and bordering are genetically complex, with multiple contributing quantitative trait loci (QTLs), and refine one QTL to identify a new gene affecting the *daf-7* pathway, the GABA receptor EXP-1.

## Results

### At least five loci that differ between N2 and CB4856 affect social behavior

**Figure 1:**
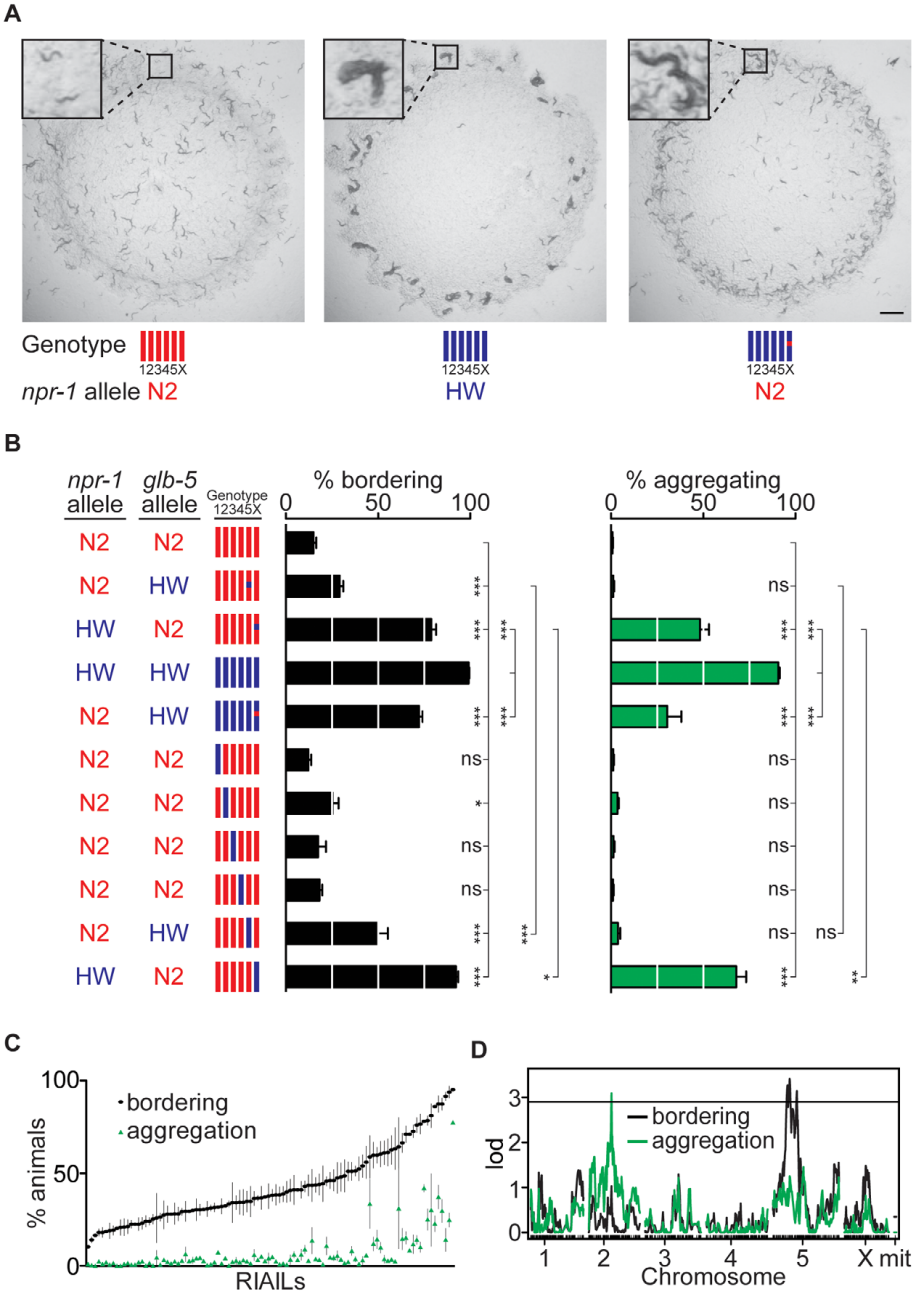
Identification of QTLs for social behavior. (A) Behavior of N2 animals (left), HW animals (middle), and HW animals with the N2 *npr-1* allele (right). Photographs were taken three days after three adult hermaphrodites produced self-progeny on plates seeded with *E. coli* OP50. Scale bar, 2 mm. Insets show individual solitary N2 animals, a group of aggregating HW animals, and aggregating and non-aggregating animals from the HW strain with the N2 *npr-1* allele. For genotypes in all figures, red denotes N2 DNA, blue denotes HW DNA. (B) Bordering and aggregation behaviors of *npr-1* near-isogenic lines (NILs) and of chromosome-substitution strains. In this and other figures, bordering and aggregation were measured on 150 adult animals two hours after transferring to *E. coli* OP50 seeded plates (see Methods); values represent the mean of at least three assays per strain. Error bars, s.e.m. * *P*<0.05, ** *P*<0.01, *** *P*<0.001, by ANOVA with Bonferroni test. ns, not significant. (C) Behaviors of 102 N2-HW recombinant inbred advanced intercross lines (RIAILs) that carry the N2 *npr-1* allele. (D) QTL analysis of RIAILs shown in (C). The horizontal line denotes the *P*<0.05 genome-wide significance threshold. lod, log likelihood ratio.

The Hawaiian CB4856 (HW) *C. elegans* strain is highly divergent from the N2 laboratory strain at many loci [31], including the neuropeptide receptor gene *npr-1*. To probe the combined effects of loci other than *npr-1* on aggregation and bordering behavior, we examined two near-isogenic lines (NILs) that differed from each parental strain by the genetic substitution of a small region near the *npr-1* gene (Figure 1A, 1B; see Methods). As shown previously, a NIL in which the HW allele of *npr-1* was introduced into an N2 background resulted in increased levels of aggregation and bordering [18], [24] (Figure 1B). These levels were, however, significantly lower than those of the HW strain under the conditions examined. Conversely, introducing the N2 allele of *npr-1* into the HW background did not restore behavior to N2-like levels, instead resulting in a strain with intermediate levels of aggregation and bordering (Figure 1A, 1B). These results indicate that loci in addition to *npr-1* affect aggregation and bordering behaviors.

To systematically define genetic differences between N2 and HW, we characterized chromosome substitution strains (CSS) in which each of the six N2 chromosomes was individually replaced by a HW chromosome [32]. Strains bearing HW chromosomes II or V had significantly higher bordering than N2, and the strain bearing the HW X chromosome had high levels of both bordering and aggregation, identifying three chromosomes with loci affecting these behaviors (Figure 1B). The known laboratory-derived mutations in *npr-1* and *glb-5* are on X and V, respectively. However, the effects of X chromosome substitution were significantly greater than those of the HW *npr-1* NIL, and effects of chromosome V substitution were significantly greater than those of a similar NIL bearing the HW allele of *glb-5*. These results imply the existence of at least one additional QTL on each of chromosomes V and X (Figure 1B). Thus the combination of CSS and NIL analysis indicates that aggregation and bordering are affected by at least five loci that differ between N2 and HW: one or more loci on II, *glb-5* and at least one additional locus on V, and *npr-1* and at least one additional locus on X.

In a parallel approach, QTL analysis was performed on recombinant inbred advanced intercross lines (RIAILs) derived from crosses between N2 and HW [31]. To set aside the large effect of the *npr-1* mutation, we examined only strains with the N2 allele of *npr-1*. These 102 RIAILs had a continuous quantitative distribution of bordering and aggregation behaviors, implying the existence of multiple QTLs rather than one locus of large effect (Figure 1C). Bordering and aggregation behaviors were strongly but not perfectly correlated in the RIAILs (r = 0.73, 99%C.I. = 0.58–0.83), suggesting that genetic contributions to bordering and aggregation in these strains are similar but perhaps not identical.

QTL analysis of the RIAILs identified a significant QTL on chromosome II (II-QTL) for aggregation and a significant QTL on chromosome V (V-QTL) for bordering (Figure 1D). The II-QTL explains 8.2% (*P*<0.01) of the aggregation variance and 5.3% (*P* = 0.019) of the bordering variance in the RIAILs, whereas the V-QTL explains 14% (*P*<0.01) of the bordering variance and an insignificant fraction of the aggregation variance (see Methods). The chromosome V QTL overlaps *glb-5*, as well as covering a broader region that may encompass the second bordering QTL inferred from the chromosome V substitution strain (Figure 1B, 1D). The II-QTL does not correspond to a previously characterized locus, and was analyzed further.

### A QTL for aggregation and bordering maps to a 6.2 kb interval

**Figure 2:**
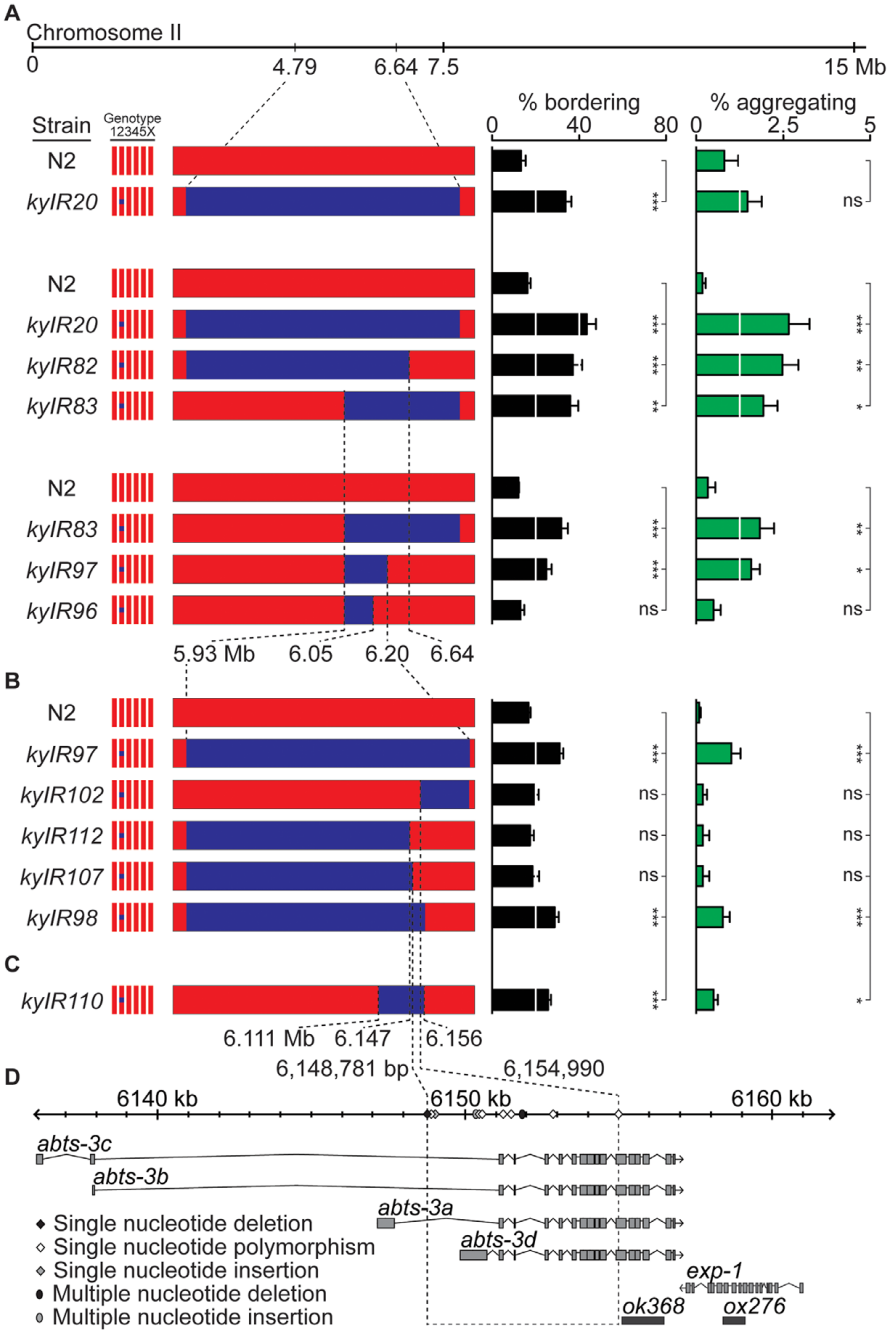
A social behavior II-QTL maps to a 6.2 kb region. (A) Bordering and aggregation behaviors of recombinants in the II-QTL region introduced as NILs into an N2 background. (B) Behaviors of NILs derived from *kyIR97*. (C) Behaviors of *kyIR110*, a near-isogenic line containing 45 kb of HW DNA in an N2 background. (D) Expansion of the 6.2 kb QTL, showing polymorphisms between N2 and HW, location of transcripts (see Methods), and location of deletion alleles used in Figure 3 and Figure 4. Error bars, s.e.m. * *P*<0.05, ** *P*<0.01, *** *P*<0.001 by t-test or ANOVA with Dunnett test. ns, not significant.

The QTL analysis of RIAILs placed the II-QTL near 6.62 Mb (1.5-LOD support interval = 2.48–9.55 Mb). To confirm this map position, we created a NIL, *kyIR20*, containing the peak of the HW II-QTL (from 4.77 to 6.65 Mb) in an N2 background. The behavior of *kyIR20* resembled that of the chromosome substitution strain bearing all of HW chromosome II, with 2.5-fold more bordering than the N2 strain, and a small effect on aggregation (Figure 2A). The effects of the II-QTL on bordering and aggregation cosegregated throughout fine-mapping of the QTL, suggesting that they have a common genetic basis (see below).

To identify the specific HW region(s) that affect behavior, we generated recombinants between N2 and the *kyIR20* NIL, deriving NILs with less HW DNA than *kyIR20*. These NILs defined an interval from 6.047 to 6.192 Mb, a region encompassing 56 genes, as a minimal region sufficient to promote bordering and aggregation (Figure 2A). A NIL that behaved significantly different from N2, *kyIR97*, contained only 270 kb of HW DNA (Figure 2A). To fine-map this II-QTL, 5000 F2 progeny of crosses between the *kyIR97* NIL and N2 were screened at the DNA level for recombination events within the QTL interval, and the recombinants were tested for aggregation and bordering behaviors. Five informative recombination events split this region. The behavior of the five recombinants indicated the presence of genetic changes necessary for bordering and aggregation in a 6.2 kb interval (6,148,781–6,154,990) (Figure 2B, 2D). *kyIR110*, a NIL with less than 45 kb of HW DNA covering the 6.2 kb interval, retained higher bordering and aggregation behaviors than N2 (Figure 2C). These genetic results definitively placed a QTL within the 45 kb associated with *kyIR110*, and support a location for one quantitative trait nucleotide within the smaller 6.2 kb interval. However, a trend toward smaller behavioral effects as the QTL was refined leaves open the possibility that additional causative polymorphisms between N2 and HW are present in the larger *kyIR20* interval.

The 6.2 kb minimal QTL interval fell within a single gene, *abts-3*, which encodes a predicted anion transporter (Figure 2D). Sequencing this region uncovered 11 polymorphisms between HW and N2 (Figure 2D and Table S1): five noncoding single nucleotide polymorphisms (SNPs), two coding SNPs (*abts-3a* G615D, *abts-3d* Q118P), one single nucleotide deletion, one single nucleotide insertion, a three-nucleotide single amino acid insertion (*abts-3d* 115I116), and a 23-nucleotide deletion in HW. Sequence analysis of 59 additional wild strains indicated that 10 of the 11 sequence polymorphisms were represented in other wild *C. elegans* populations, whereas one SNP was present only in HW (Table S1 and data not shown). Notably, the N2 sister strain LSJ2, which separated from N2 soon after their isolation from the wild [24], was identical in sequence to N2 at this locus. Thus the N2 sequences are likely to be present in wild populations, and not laboratory-derived. The existence of one private polymorphism in HW is consistent with the fact that HW is one of the most divergent wild *C. elegans* strains, harboring many polymorphisms not found in any other strain [33].

### The GABA receptor *exp-1* is a Quantitative Trait Gene affected by the II-QTL

The mapping of the II-QTL defined the location of the relevant sequence change, but not necessarily the affected gene, as noncoding regulatory changes could act at a distance to affect neighboring genes [34]–[38]. To define the gene affected by the II-QTL, we performed quantitative complementation tests between the II-QTL and loss-of-function mutations in genes in the region [1], [39]. In this test, the N2 and HW QTLs were examined as heterozygotes with null alleles of candidate genes, with the expectation that the null allele would fail to complement the QTL with reduced activity.

**Figure 3:**
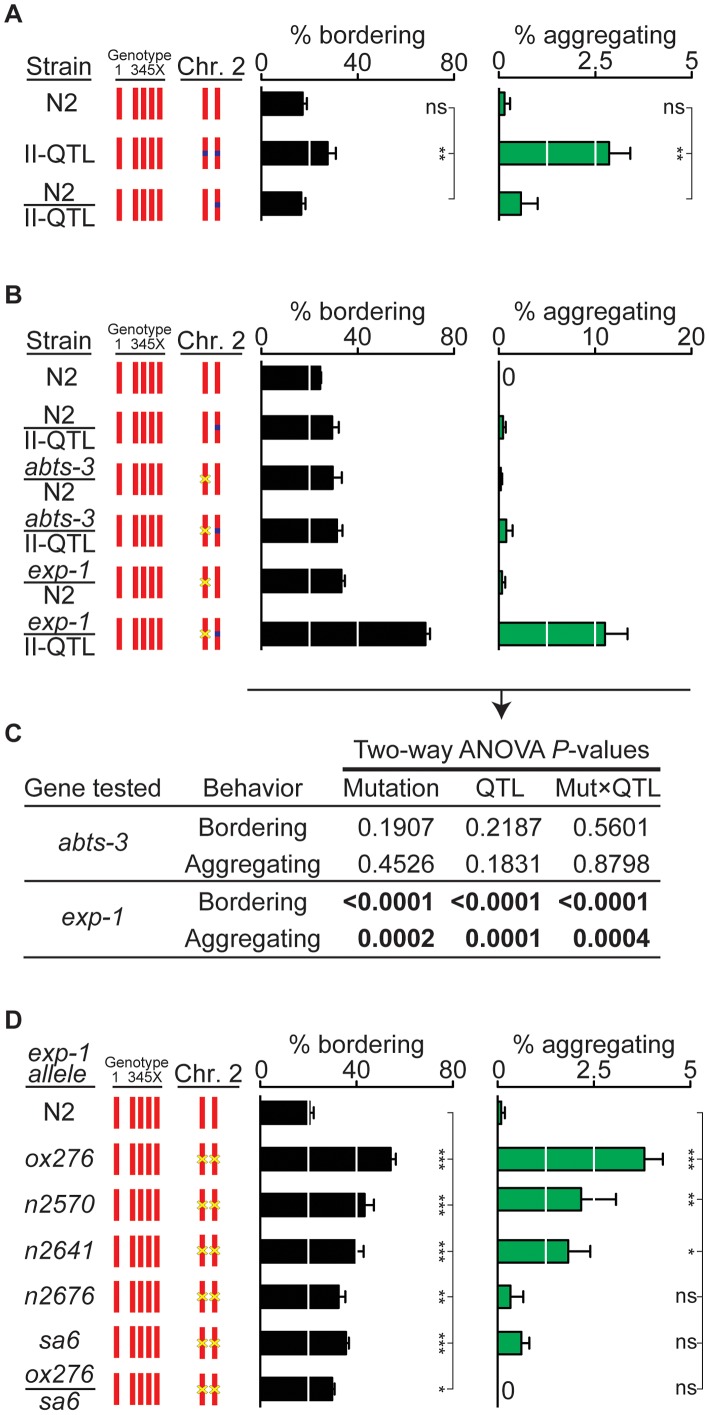
*exp-1* is a quantitative trait gene for bordering and aggregation. (A) Dominance test between N2 and the II-QTL (n = 7). (B) Quantitative complementation tests between the HW II-QTL, N2, and deletion mutants *abts-3(ok368)* and *exp-1(ox276)*. Heterozygote F1 progeny from crosses were identified using a fluorescent marker (n = 6 assays for each cross). Yellow ‘x’ denotes a deletion mutation. A transgenic fluorescent marker used to identify F1s in this cross elevated bordering slightly and may also accentuate aggregation (see Figure S4). (C) Analysis of variance of data in panel (B). Two-way ANOVA used Mutation (N2 vs mutant allele) and QTL (N2 vs II-QTL) as variables and was performed for both *abts-3(ok368)* and *exp-1(ox276)* mutations. A significant *P* value for the Mut×QTL interaction indicates failure to complement, a defining feature of quantitative trait genes [1], [39]. (D) Behaviors of *exp-1(ox276)*, a deletion allele, *exp-1(n2570)*, *exp-1(n2641)*, *exp-1(n2676)*, *and exp-1(sa6)* missense alleles, and *exp-1(ox276)/exp-1(sa6)* trans-heterozygotes. Yellow ‘x’ denotes a mutation. Error bars, s.e.m. In (A) and (D), * *P*<0.05, ** *P*<0.01, *** *P*<0.001 by ANOVA with Dunnett test. ns, not significant.

Initial experiments indicated that the bordering and aggregation behaviors of an N2 II-QTL/HW II-QTL heterozygote resembled the N2 II-QTL homozygote (Figure 3A). The recessive nature of the HW II-QTL suggested that the HW phenotype should be observed in a HW/null heterozygote. Since the 6.2 kb minimal QTL interval was fully contained within *abts-3*, this gene was considered the most promising candidate. However, a deletion allele of *abts-3(ok368)* complemented the HW II-QTL, as well as the N2 II-QTL, to give N2-like behavior in heterozygotes (Figure 3B; the location of the mutation is shown in Figure 2D). This result argued against *abts-3* being the gene affected by the II-QTL.

A second gene close to the II-QTL is *exp-1*, which encodes a γ-aminobutyric acid (GABA)-gated cation channel [40]; the stop codon of *exp-1* is 2.2 kb away from the 6.2 kb QTL (Figure 2D). In a quantitative complementation test, a loss-of-function mutation in *exp-1(ox276)* failed to complement the HW II-QTL, with the heterozygote showing substantial bordering and aggregation behaviors (Figure 3B). Control experiments demonstrated that the *exp-1* mutation was fully complemented by the N2 II-QTL, excluding dominant effects of *exp-1* (Figure 3B). Two-way ANOVA provided strong statistical support for an interaction between the II-QTL and *exp-1*, but not *abts-3* (Figure 3C). These results suggest that *exp-1* is a quantitative trait gene that affects bordering and aggregation.

To explain these results, we suggest that noncoding variation 3′ of the *exp-1* transcript, within the *abts-3* gene, modifies aggregation and bordering behavior (at least in part) by affecting the activity of *exp-1*. The overall abundance of *exp-1* mRNA measured by quantitative RT-PCR was similar in N2 and in the HW II-QTL strain (Figure S1), suggesting that the 3′ sequences in the II-QTL may confer specific spatial or temporal patterns of expression, rather than affecting total mRNA levels.

### EXP-1 and the neurotransmitter GABA regulate social behavior

As an independent test of the role of *exp-1* in social behavior, we examined five *exp-1* mutant alleles in an N2 genetic background: a predicted null allele, *exp-1(ox276)*, and four missense alleles, *exp-1(n2570)*, *exp-1(n2641)*, *exp-1(n2676)*, and *exp-1(sa6)* [40]. All *exp-1* mutants had high levels of bordering behavior, and *exp-1(ox276)*, *exp-1(n2570)*, and *exp-1(n2641)* had significantly increased aggregation (Figure 3D). In a complementation test, *exp-1(ox276)/exp-1(sa6)* trans-heterozygotes failed to complement for bordering behavior, as expected if they affect the same complementation group (gene) (Figure 3D). These results indicate that normal *exp-1* activity suppresses bordering and aggregation in the N2 strain, and suggest that the II-QTL from HW has a reduced level of *exp-1* activity.

Previous studies of *exp-1* in the enteric nervous system defined a region sufficient for rescue of *exp-1* phenotypes related to defecation [40], but this clone did not include the 6.2 kb downstream region defined by the II-QTL. A transgene containing the minimal *exp-1* region involved in defecation failed to rescue the bordering and aggregation defects of the *exp-1(ox276)* deletion mutant (Figure S2, transgene ‘6’), suggesting that it lacked regulatory sequences for *exp-1* expression relevant to the bordering phenotype. Among multiple tested transgenes spanning the *exp-1* locus and adjacent sequences, only transgenes covering 70 kb encompassing both *exp-1* and *abts-3* effectively rescued the social behaviors of *exp-1(ox276)* (Figure S2, transgene ‘4+5’). Similar transgenes reduced bordering in the HW II-QTL strain (Figure S2). These results suggest that *exp-1* regulation of aggregation and bordering requires long-range regulation, including substantial sequences 3′ of *exp-1* (see Discussion).

**Figure 4:**
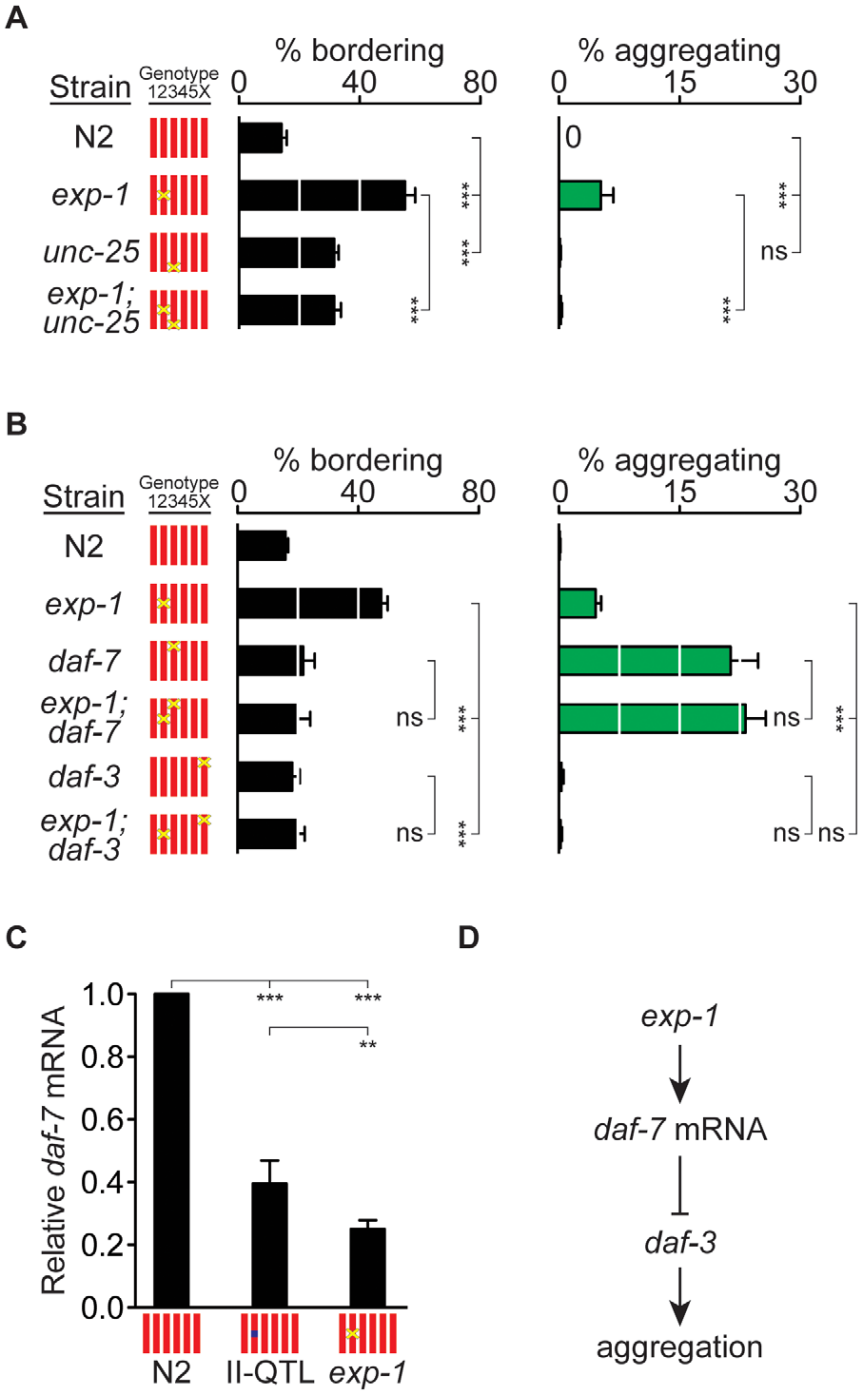
*exp-1* acts with GABA and *daf-7* TGF-β to regulate bordering and aggregation. (A) Behaviors of *exp-1(ox276)*, *unc-25(n2324)*, and double mutants. (B) Behaviors of *exp-1(ox276)*, *daf-7(e1372)*, *daf-3(e1376)*, and double mutants. (C) Relative amounts of *daf-7* mRNA in N2, HW II-QTL *kyIR110* near-isogenic line, and *exp-1(ox276)*, measured by quantitative RT-PCR, (D) Model for *exp-1* effects on aggregation. In (A) and (B), error bars, s.e.m. *** *P*<0.001 by ANOVA with Bonferroni test. ns, not significant. In (C), error bars, 95% C.I. ** *P*<0.01, *** *P*<0.001 by t-tests with Bonferroni correction.

*exp-1* encodes an unconventional GABA-gated cation channel that depolarizes cells in the presence of GABA [40]; it is one of four genetically characterized *C. elegans* GABA receptors. All GABAergic signaling in *C. elegans* is blocked by mutations in *unc-25* [41], [42], which encodes the GABA biosynthetic enzyme glutamate decarboxylase, so *unc-25* mutants would be expected to have phenotypes related to those of *exp-1*. Indeed, *unc-25(n2324)* mutants showed bordering behaviors, albeit weaker than those of the *exp-1* deletion mutant (Figure 4A). An *exp-1(ox276); unc-25(n2324)* double mutant did not have enhanced defects, and in fact resembled the milder *unc-25* mutant rather than the stronger *exp-1* mutant (Figure 4A). The shared effects of *exp-1* and *unc-25* are consistent with the hypothesis that GABA regulates bordering behavior by activating *exp-1*. The milder phenotype of *unc-25* and the double mutants suggests that GABA might also activate a second GABA receptor with an opposite effect to *exp-1*.

### *exp-1* acts in the *daf-7* TGF-β pathway

The *daf-7* neuroendocrine pathway, which is regulated by food and pheromone levels, inhibits aggregation in the N2 genetic background [20]. In the standardized aggregation assay used here, *daf-7(e1372)* mutant adults showed high levels of aggregation and low levels of bordering compared to *exp-1* mutants (Figure 4B). Adult *exp-1(ox276); daf-7(e1372)* double mutant animals did not have enhanced behavioral phenotypes compared to single mutants, suggesting that the two genes act in a common pathway (Figure 4B). In agreement with this possibility, a mutation in the downstream *daf-3* co-SMAD transcriptional regulator, which suppresses *daf-7* aggregation phenotypes [20], also suppressed the bordering and aggregation of *exp-1* (Figure 4B).

The relatively low levels of bordering in *daf-7* adults were unexpected based on the literature [20], but may be explained by developmentally-regulated differences in behaviors: we observed that L4-stage *daf-7* animals bordered and aggregated more extensively than adult animals (Figure S3), and previous studies did not analyze these stages separately. In L4 animals, as in adults, the bordering and aggregation of *exp-1(ox276); daf-7(e1372)* double mutant animals was no stronger than that of the most severe single mutant, suggesting participation in a common pathway (Figure S3).

Transcription of *daf-7* is regulated by environmental conditions: *daf-7* mRNA levels increase when food is abundant and decrease in the presence of density pheromones [26], [27]. This transcriptional regulation is partly intrinsic to the ASI sensory neuron that expresses *daf-7*, and partly determined by intercellular signaling between other sensory neurons and ASI [43]. Using RT-PCR, we asked whether *daf-7* transcriptional regulation was altered in *exp-1* mutants. *exp-1(ox276)* mutant animals had a 4-fold decrease in *daf-7* mRNA levels compared to controls (Figure 4C). Moreover, *daf-7* mRNA levels in the minimal II-QTL strain *kyIR110* were intermediate between those of N2 and those of *exp-1* mutants, consistent with the possibility that the II-QTL causes a partial reduction of *exp-1* activity (Figure 4C). In combination with the genetic epistasis results, these observations suggest that *exp-1* and the II-QTL stimulate aggregation and bordering at least partly through effects on *daf-7* expression (Figure 4D).

## Discussion

Using chromosome substitution strains and RIAILs between N2 and HW *C. elegans* strains, we found that at least five QTLs affect bordering and aggregation behavior. Two QTLs correspond to the known laboratory-acquired mutations in *npr-1* and *glb-5*, two remain to be identified, and one QTL maps to a region near the *abts-3* and *exp-1* genes. The minimal 6.2 kb QTL is entirely contained within *abts-3*, but our results argue that the transcription unit affected by the QTL is the neighboring gene *exp-1*, which encodes a GABA receptor. *exp-1* and *abts-3* genes are adjacent and convergently transcribed in all five *Caenorhabditis* species for which data is available (www.wormbase.org), suggesting that this synteny might be functionally relevant.

Both association studies and linkage studies map causal genetic variants (i.e. functionally relevant sequence polymorphisms), but if the causal variants affect regulatory sequences, they can be located far from the affected gene or genes [1], [38]. The *exp-1* transcript ends 2.2 kb from the nearest polymorphism in the II-QTL, suggesting that the QTL affects a regulatory region 3′ to the *exp-1* coding region. Most known *C. elegans* regulatory sites are upstream of or within coding regions, but there are precedents for 3′ transcriptional regulatory elements, including an element located 5.6 kb 3′ of the *egl-1* cell death gene [37] and elements located 3′ to the *osm-9* and *ocr-2* family of sensory ion channel genes [44], [45], and there are also many mammalian precedents for 3′ regulation [46], [47]. In the context of natural variation, a recent study identified a 3′ regulatory region of the *Nasonia unpaired-like* gene that affects wing width differences between wasp species [48]. 3′ regulatory regions were not represented in over 300 other genetic variants that cause phenotypic variation within and between species; this may represent either a real difference or an ascertainment bias toward 5′ regulatory elements (studies compiled in [49], [50]). Human SNPs that affect gene expression (eQTNs) are three times more abundant 5′ of transcriptional start sites than 3′ of transcriptional end sites, and human enhancers are also more commonly 5′ of the gene they regulate, suggesting that the bias toward 5′ regulatory elements is real but not absolute [51], [52].

Most *C. elegans* mutations can be rescued by transgenes that cover relatively short regions surrounding the gene of interest. By contrast, the aggregation and bordering behaviors of the II-QTL and the *exp-1(ox276)* mutant were refractory to rescue with small transgenes that could rescue the enteric nervous system defects of the *exp-1* mutation [40]. The only genomic DNA fragments that successfully rescued *exp-1(ox276)* aggregation and bordering covered 26 kb 5′ and 40 kb 3′ of the *exp-1* coding region, including all *abts-3* transcripts and several additional transcripts. One possible explanation for this result is that appropriate *exp-1* expression requires several long-range *cis-*regulatory elements, including the 6.2 kb QTL and other distal sequences. A second possibility is that *exp-1* is unusually sensitive to general DNA context, resembling genes expressed in the *C. elegans* germline, which are transcriptionally silenced unless they are embedded in a complex genomic context [53].

*exp-1* encodes an unconventional GABA-gated cation channel that depolarizes cells rather than inhibiting them [40]. It was first identified based on its excitatory action on enteric muscles, but *exp-1–*GFP fusions are also expressed in some neurons (PDA, RID, ADE, SABD) [40]. The relevant site of *exp-1* expression for social behavior is unknown, because the minimal transgene that rescues the enteric defect did not rescue social behavior, and therefore may not encompass the full expression pattern of *exp-1*. A fuller characterization of the 70 kb genomic region that does rescue social behavior may provide insights into sites of *exp-1* expression. GABA-deficient animals have mild bordering and aggregation behavior phenotypes, consistent with a likely function of *exp-1* as a GABA receptor. GABA is produced by just 26 neurons in *C. elegans;* identification of the relevant GABAergic neurons may assist in defining the *exp-1* circuit.

*exp-1* and the *daf-7* TGF-β pathway show genetic interactions, suggesting a common role in aggregation. In agreement with this interaction, the level of *daf-7* mRNA was reduced in *exp-1* null mutants and in animals with the HW *exp-1* QTL. *exp-1* may be part of the system for detecting environmental stresses that regulates *daf-7* expression and neuroendocrine function. The *daf-7* pathway and the GABA neurotransmitter system also cooperate to regulate *C. elegans* dauer development [54], perhaps by using the same transcriptional mechanism defined here.

These results add to evidence that genetic polymorphisms affecting neurotransmitter receptors are sources of natural behavioral variation, and reinforce the importance of studying natural variation as a means toward new biological insights. In humans, very few genetic variants that affect behavioral traits have been mapped. Among these are the ligand-gated ion channels CHRNA3 and CHRNA5, which encode nicotinic acetylcholine receptor subunits implicated in cigarette smoking behavior by genome-wide association studies [55]. Thus ligand-gated ion channels represent sites of behavioral variation both in *C. elegans* and in humans.

Previous studies in *C. elegans* and in humans have suggested that G protein-coupled neurotransmitter receptors may be preferred sites of behavioral variation. Among the G protein-coupled receptors implicated in behavioral variation are the *C. elegans* tyramine receptor *tyra-3*, which modifies exploratory behavior [56], the *C. elegans* neuropeptide receptor *npr-1*, which affects social behavior [18], and human variation in receptors for serotonin, dopamine, and several neuropeptides that has been associated with psychiatric traits [57]–[60]. Both G protein-coupled receptors and nicotinic acetylcholine receptors in the brain are considered modulatory because they are not essential for fast neurotransmission [61], and in both cases the receptors belong to large families of related genes that could provide a reservoir for genetic variation. These properties may allow polymorphisms in modulatory receptors to generate variation in neuronal circuits while sparing the core functions of the nervous system. Our results demonstrate the ability to identify such genetic variants even when they have small quantitative effects on behavior.

## Methods

### Nematode growth

Strains were grown and maintained under standard conditions at 22–23°C (room temperature) on Nematode Growth Medium (NGM) 2% agar plates [62]. All animals used for behavioral assays were grown on plates seeded with *Escherichia coli* OP50.

### Behavioral assays

Aggregation and bordering behaviors were measured essentially as described [18], with modifications from [19]. Briefly, 2–3 week old 2% agar NGM plates (stored at 4°C) were seeded with 200 µL of a saturated *E. coli* OP50 bacterial culture in LB 2 days before the assay and left at room temperature. 150 adult animals were picked onto the assay lawn. After two hours at 22–23°C, bordering and aggregation behavior were quantified by eye using a dissecting microscope. An animal was considered to be bordering if its whole body resided within 1 mm of the border of the bacterial lawn. Aggregation behavior was measured as the fraction of animals that were in contact with two or more other animals along at least 50% of their body length; this criterion is highly stringent but unambiguous. Each strain was tested at least five times, except for CB4856 (Figure 1B, four assays), RIAILs (Figure 1C, at least three assays each), and transgenic rescued lines (Figure S3, at least three assays per line, and at least three lines per tested clone). Introgression lines (Figure 2) were tested at least seven times.

### Quantitative trait locus analysis

The N2-HW recombinant inbred advanced intercross lines (RIAILs) used in this study represent the terminal generation of a 20-generation pedigree founded by reciprocal crosses between N2 and HW. The lines were constructed through 10 generations of intercrossing followed by 10 generations of selfing [31]. They have been genotyped at 1454 nuclear and one mitochondrial markers and have a 5.3-fold expansion of the F2 genetic map [31]. Each RIAIL was tested at least three times. QTL analysis was performed on the mean bordering and aggregation of N2-HW RIALs by nonparametric interval mapping at 1 cM intervals in R/qtl [63]. Significance levels were estimated from 10,000 permutations of the data.

Percent variance explained by QTLs was based on QTLs defined by the marker with the highest LOD score in the II-QTL (which reached genome-wide significance for aggregation) and the V-QTL (which reached genome-wide significance for bordering). Percent variance explained was calculated for each QTL with ANOVA. While it is not common to measure effect sizes for traits for which a statistically significant QTL is not found, we report the effect sizes of the II-QTL and the V-QTL for both bordering and aggregation behaviors in the text because the two traits have a strong genetic correlation.

### Generation of near-isogenic lines

Near-isogenic lines were created by backcrossing a chromosomal region or allele into the desired genetic background as described below. Desired segments were then inbred to homozygosity. Marker positions are based on Wormbase release WS229.

CX11922 *(CB4856>N2) kyIR20* II: QX111, a RIAIL containing the HW II-QTL was backcrossed to *clr-1 dpy-10* (in an N2 background) for 9 generations, picking non-Clr, non-Dpy males each generation. The introgression breakpoints are, on the left, between 4,783,398 (indel) and 4,800,876 (marker haw25011), and on the right, between 6,627,080 (marker haw25929) and 6,672,356 (marker haw25938).

CX13072 *(CB4856>N2) kyIR82* II: CX11922 *kyIR20* was crossed to N2 and an F2 recombinant was made homozygous. The introgression breakpoints are, on the left, between 4,783,398 (indel) and 4,800,876 (marker haw25011), and on the right, between 6,215,940 (marker haw25805) and 6,442,763 (indel).

CX13073 *(CB4856>N2) kyIR83* II: CX11922 *kyIR20* was crossed to N2 and an F2 recombinant was made homozygous. The introgression breakpoints are, on the left, between 5,926,596 (indel) and 5,941,581 (indel), and on the right, between 6,627,080 (marker haw25929) and 6,672,356 (marker haw25938).

CX13602 *(CB4856>N2) kyIR97* II: CX13073 *kyIR83* was crossed to N2 and an F2 recombinant was made homozygous. The introgression breakpoints are, on the left, between 5,926,596 (indel) and 5,941,581 (indel), and on the right, between 6,195,603 (marker haw25802) and 6,198,696 (marker haw25803).

CX13601 *(CB4856>N2) kyIR96* II: CX13073 *kyIR83* was crossed to N2 and an F2 recombinant was made homozygous. The introgression breakpoints are, on the left, between 5,926,596 (indel) and 5,941,581 (indel), and on the right, between 6,047,397 (marker haw25707) and 6,064,898 (marker haw25718).

### Fine-mapping of the II-QTL for social behavior

N2 animals were crossed to CX13602 *kyIR97*, F1 hermaphrodites were selfed and 5,000 individual F2 hermaphrodites were dispensed into single wells of 96-well plates with the use of a worm sorter (COPAS Biosort system; Union Biometrica). These F2s were grown on 200 µL of an *E. coli* OP50 suspension in S-basal buffer with cholesterol, rotating at 230 RPM, at 22°C for 6 days (1–2 generations). The progeny of each F2 were genotyped at markers 5,941,581 (indel) and 6,195,603 (marker haw25802), a 0.41 cM interval, and recombinants between these markers were identified. Animals homozygous for the recombinant chromosome were then tested for social behavior.

CX13854 *(CB4856>N2) kyIR102* II: The left breakpoint fell at 6,155,430 (marker haw25773); the breakpoint on the right is between 6,195,603 (marker haw25802) and 6,198,696 (marker haw25803).

CX14013 *(CB4856>N2) kyIR112* II: The introgression breakpoints are, on the left, between 5,926,596 (indel) and 5,941,581 (indel), and on the right, between 6,132,624 (marker haw25748) and 6,146,765 (marker haw25767).

CX14008 *(CB4856>N2) kyIR107* II: The introgression breakpoints are, on the left, between 5,926,596 (indel) and 5,941,581 (indel), and on the right, between 6,147,008 (marker haw25768) and 6,148,900 (marker haw25769).

CX13845 *(CB4856>N2) kyIR98* II: The introgression breakpoints are, on the left, between 5,926,596 (indel) and 5,941,581 (indel), and on the right, between 6,156,160 (marker haw25774) and 6,156,620 (marker haw25775).

CX14011 *(CB4856>N2) kyIR110* II: The introgression breakpoints are, on the left, between 6,111,057 (marker haw25735) and 6,117,083 (marker haw25738), and on the right, between 6,156,160 (marker haw25774) and 6,156,620 (marker haw25775).

### Identification of the *abts-3d* isoform

The *abts-3d* isoform was not present in the standard Gene Models of Wormbase (WS229). The existence of an alternative first exon in *abts-3d* was inferred from the following evidence: (1) presence of Illumina sequence reads from cDNA derived from polyA+ RNA of larval and adult *C. elegans* covering the putative exon, including reads that span an inferred 3′ exon junction but absence of 5′ exon junction reads (as shown in Wormbase), (2) presence of a long open reading frame that overlaps the cDNA reads, (3) high degree of conservation of the putative exon with other *Caenorhabditis* species, relative to other introns in the gene, (4) high evolution rate across *Caenorhabditis* species of third positions of putative codons in the region relative to first and second positions, (5) presence of a 3-bp indel in the region, an unusual feature in non-coding sequences.

### Quantitative RT–PCR

Animals were synchronized in the L1 stage (16 to 20 h post-egg laying) by allowing adults to lay eggs on seeded plates for four hours. The L1 stage was chosen because (1) it is an important time point for *daf-7* regulation, (2) neuronal genes are expressed at the highest relative level with respect to total RNA in L1 animals, and (3) it is easy to maintain tight developmental synchrony at this stage. Total RNA from these L1 stage synchronized cultures was isolated with Trizol-chloroform, precipitated with an equal volume of 70% ethanol and cleaned with Zymo Quick-RNA MicroPrep according to the manufacturer's instuructions. 800 ng of RNA and oligo-dT were used for reverse transcription using SuperScript III First-Strand Synthesis (Invitrogen) according to the manufacturer's instructions. Real-time PCR was performed with Fast SYBR Green Master Mix (Applied Biosystems) on a 7900HT Real-Time PCR System (Applied Biosystems). *cdc-42* was used as the calibrator for relative quantitation [64]. Primers used were:

*exp-1_F*, ttttggcagatttcaacagc

*exp-1_R*, ttcatcattttcctccatcaag

*daf-7_F*, gcaccaactcaggtgtttgtat

*daf-7_R*, aatccctttggtgcctcttt

*cdc-42_F*, cggatgttggagagaagttgg

*cdc-42_R*, ctgttgtggtgggtcgagag

### Extrachromosomal transgenes

Transgenes were made by injection of DNA clones into the gonads of young adult hermaphrodites together with a fluorescent coinjection marker [65]. To control for variation among transgenes, at least three independent lines from each injection were characterized.

The following fosmids were injected alone or in combination, as described below in Transgenic strains: WRM066cE09, WRM0620bF02, WRM0610bG09, H35N03, WRM0612dD07. Prior to injection, fosmid structures were confirmed by restriction digest with diagnostic enzymes.

pAB05 is a 7.6 kb genomic *exp-1 NsiI/ScaI* fragment that contains an in-frame GFP fusion in the intracellular loop between M3 and M4 of EXP-1 [40].

### Strains

#### “Wild-type” strains

Strain – Origin:

AB1 – Adelaide, Australia

CB4853 – Altadena, California, USA

CB4856 (HW) – Hawaii, USA

JU258– Madeira, Portugal

LSJ2 – Bristol, England

MY1 – Lingen, Germany

MY10 – Roxel, Germany

MY14 – Mecklenbeck, Germany

MY16 – Mecklenbeck, Germany

MY18 – Roxel, Germany

N2 – Bristol, England

QX1216 – San Francisco, California, USA

#### N2-HW RIAILs for bordering and aggregation QTL analyses

QX1, QX3, QX4, QX5, QX6, QX8, QX9, QX15, QX16, QX17, QX18, QX20, QX25, QX26, QX27, QX29, QX32, QX33, QX38, QX43, QX44, QX47, QX48, QX49, QX51, QX52, QX53, QX54, QX55, QX56, QX57, QX62, QX68, QX70, QX71, QX72, QX73, QX74, QX76, QX78, QX79, QX80, QX81, QX82, QX83, QX84, QX85, QX87, QX90, QX92, QX93, QX94, QX95, QX96, QX97, QX99, QX100, QX102, QX103, QX110, QX112, QX114, QX115, QX120, QX121, QX128, QX129, QX137, QX140, QX147, QX156, QX157, QX161, QX163, QX165, QX171, QX174, QX175, QX176, QX177, QX178, QX181, QX186, QX187, QX189, QX190, QX192, QX193, QX194, QX203, QX206, QX210, QX212, QX216, QX217, QX218, QX220, QX221, QX227, QX228, QX230, QX231.

#### Near-isogenic lines in an N2 background

CX14180 *kyIR122 [X: ∼4.24–∼4.83 Mb, CB4856>N2]*

CX11922 *kyIR20 [II: ∼4.79–∼6.65 Mb, CB4856>N2]*

CX13072 *kyIR82 [II: ∼4.79–∼6.30 Mb, CB4856>N2]*

CX13073 *kyIR83 [II: ∼5.93–∼6.65 Mb, CB4856>N2]*

CX13602 *kyIR97 [II: ∼5.93–∼6.97 Mb, CB4856>N2]*

CX13601 *kyIR96 [II: ∼5.93–∼6.06 Mb, CB4856>N2]*

CX13854 *kyIR102 [II: ∼6.16–∼6.20 Mb, CB4856>N2]*

CX14013 *kyIR112 [II: ∼5.93–∼6.14 Mb, CB4856>N2]*

CX14008 *kyIR107 [II: ∼5.93–∼6.15 Mb, CB4856>N2]*

CX13845 *kyIR98 [II: ∼5.93–∼6.16 Mb, CB4856>N2]*

CX14011 *kyIR110 [II: ∼6.11–∼6.16 Mb, CB4856>N2]*

#### Chromosome substitution strains

WE5236 *[I, CB4856>N2]*

WE5237 *[II, CB4856>N2]*

WE5238 *[III, CB4856>N2]*

WE5239 *[IV, CB4856>N2]*

WE5240 *[V, CB4856>N2]*

WE5241 *[X, CB4856>N2]*

#### Transgenic strains

CX13765–CX13767 *kyIR97; kyEx4224–kyEx4226 [fosmid WRM066cE09 @10 ng/µL+Pelt-2::GFP @4.5 ng/µL]*

CX14588–CX14590 *kyIR110; kyEx4407, kyEx4408, kyEx4410 [fosmid WRM0620bF02 @10 ng/µL+WRM066cE09 @10 ng/µL+Pelt-2::GFP @4.5 ng/µL]*

CX14308–CX14310 *exp-1(ox276); kyEx4544–kyEx4546 [fosmid WRM0610bG09 @10 ng/µL+Pelt-2::GFP @4.5 ng/µL]*

CX14305–CX14307 *exp-1(ox276); kyEx4444, kyEx4445, kyEx4518 [fosmid H35N03 @10 ng/µL+Pelt-2::GFP @4.5 ng/µL]*

CX14585–CX14587 *exp-1(ox276); kyEx4224–kyEx4226 [fosmid WRM066cE09 @10 ng/µL+Pelt-2::GFP @4.5 ng/µL]*

CX14298–CX14300 *exp-1(ox276); kyEx4426, kyEx4428, kyEx4429 [fosmid WRM0620bF02 @10 ng/µL+Pelt-2::GFP @4.5 ng/µL]*

CX14141–CX14143, CX14145 *exp-1(ox276); kyEx4406–kyEx4408, kyEx4410 [fosmid WRM0620bF02 @10 ng/µL+WRM066cE09 @10 ng/µL+Pelt-2::GFP @4.5 ng/µL]*

CX13841–CX13844 *exp-1(ox276); kyEx4250–kyEx4253 [pAB05 @25 ng/µL+Pelt-2::mCherry @2 ng/µL]*

CX14301–CX14304 *exp-1(ox276); kyEx4431–kyEx4434 [fosmid WRM0612dD07 @10 ng/µL+pAB05 @25 ng/µL+Pelt-2::mCherry @2 ng/µL]*. A total of 16 transgenic lines were tested.

CX11693 *kyIs538 [Pglb-5::p12 hCaspase 3::SL2 GFP, Pelt-2::mCherry]*

CX13602 *kyIR97 kyIs538 [Pglb-5::p12 hCaspase 3::SL2 GFP, Pelt-2::mCherry]*

#### Mutant strains

CX13840 *abts-3(ok368) II*, autosomes outcrossed 3 times and X chromosome outcrossed completely to N2

CX13975 *exp-1(ox276) II*, outcrossed 10 times to N2

CX15377 *exp-1(n2570) II*, autosomes outcrossed 9 times and X chromosome outcrossed completely to N2

CX15378 *exp-1(n2641) II*, autosomes outcrossed 4 times and X chromosome outcrossed completely to N2

CX15379 *exp-1(n2676) II*, autosomes outcrossed 3 times and X chromosome outcrossed completely to N2

CX14520 *exp-1(sa6) II*, autosomes outcrossed 6 times and X chromosome outcrossed completely to N2

CX14453 *unc-25(n2324) III*, outcrossed 4 times to N2

CX14455 *exp-1(ox276) II; unc-25(n2324) III*

CB1372 *daf-7(e1372) III*

CX14451 *exp-1(ox276) II; daf-7(e1372) III*

CB1376 *daf-3(e1376) X*

CX14452 *exp-1(ox276) II; daf-3(e1376) X*

## Supporting Information

Figure S1 *exp-1* transcript abundance is not strongly affected by the II-QTL. Relative amounts of *exp-1* mRNA in N2 and HW II-QTL *kyIR110* near-isogenic line, measured by quantitative RT-PCR. mRNA was isolated from whole animals. Error bars, 95% C.I. ns, not significant by t-test.
(TIF)

Figure S2 Genomic transgenes can rescue bordering and aggregation in *exp-1* mutants. HW II-QTL NIL animals and *exp-1(ox276)* mutant animals were injected with N2-derived fosmids or plasmids depicted below. pAB05 is a genomic *exp-1*::GFP translational fusion that rescues the enteric defect of *exp-1* but not aggregation and bordering; it is expressed in enteric muscles and in PDA, RID, ADE, and SABD neurons. Coinjection of clones (4) and (5), spanning 70 kb, rescued aggregation and bordering. At least three independent transgenic lines were tested for each injected DNA region, with consistent results. Error bars, s.e.m. * *P*<0.05, *** *P*<0.001 by ANOVA with Dunnett tests. ns, not significant.
(TIF)

Figure S3 Genetic interactions between *exp-1* and *daf-7* in L4-stage animals. Bordering and aggregation behaviors of *exp-1(ox276)*, *daf-7(e1372)*, and double mutant L4-stage animals. Error bars, s.e.m. * *P*<0.05, ** *P*<0.01 by ANOVA with Dunnett test. ns, not significant.
(TIF)

Figure S4 The fluorescent transgenic marker used in quantitative complementation tests has a small effect on bordering. *kyIs538*, an integrated mCherry marker, was used to identify F1 cross progeny in the quantitative complementation tests in Figure 3B. Error bars, s.e.m. ** *P*<0.01 by t-test.
(TIF)

Table S1 Polymorphisms in the 6.2 kb chromosome II QTL. The sequence variants within the 6.2 kb QTL in 12 wild-type strains are reported relative to the sequence of the outgroup strain QX1216.
(TIF)
