# Supplementary figures and images for: Long-Range Regulatory Polymorphisms Affecting a GABA Receptor Constitute a Quantitative Trait Locus (QTL) for Social Behavior in Caenorhabditis elegans
Source: PLoS Genet. 2012 Dec 20;8(12):e1003157. doi: 10.1371/journal.pgen.1003157 (PMC3527333; doi:10.1371/journal.pgen.1003157)

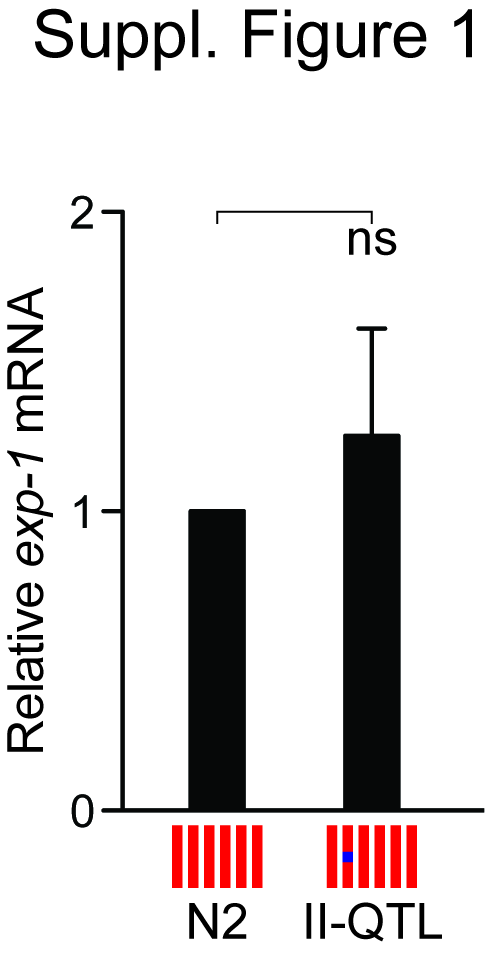

Supplement: Figure S1 — exp-1 transcript abundance is not strongly affected by the II-QTL. Relative amounts of exp-1 mRNA in N2 and HW II-QTL kyIR110 near-isogenic line, measured by quantitative RT-PCR. mRNA was isolated from whole animals. Error bars, 95% C.I. ns, not significant by t-test. (TIF) [file pgen.1003157.s001.tif]

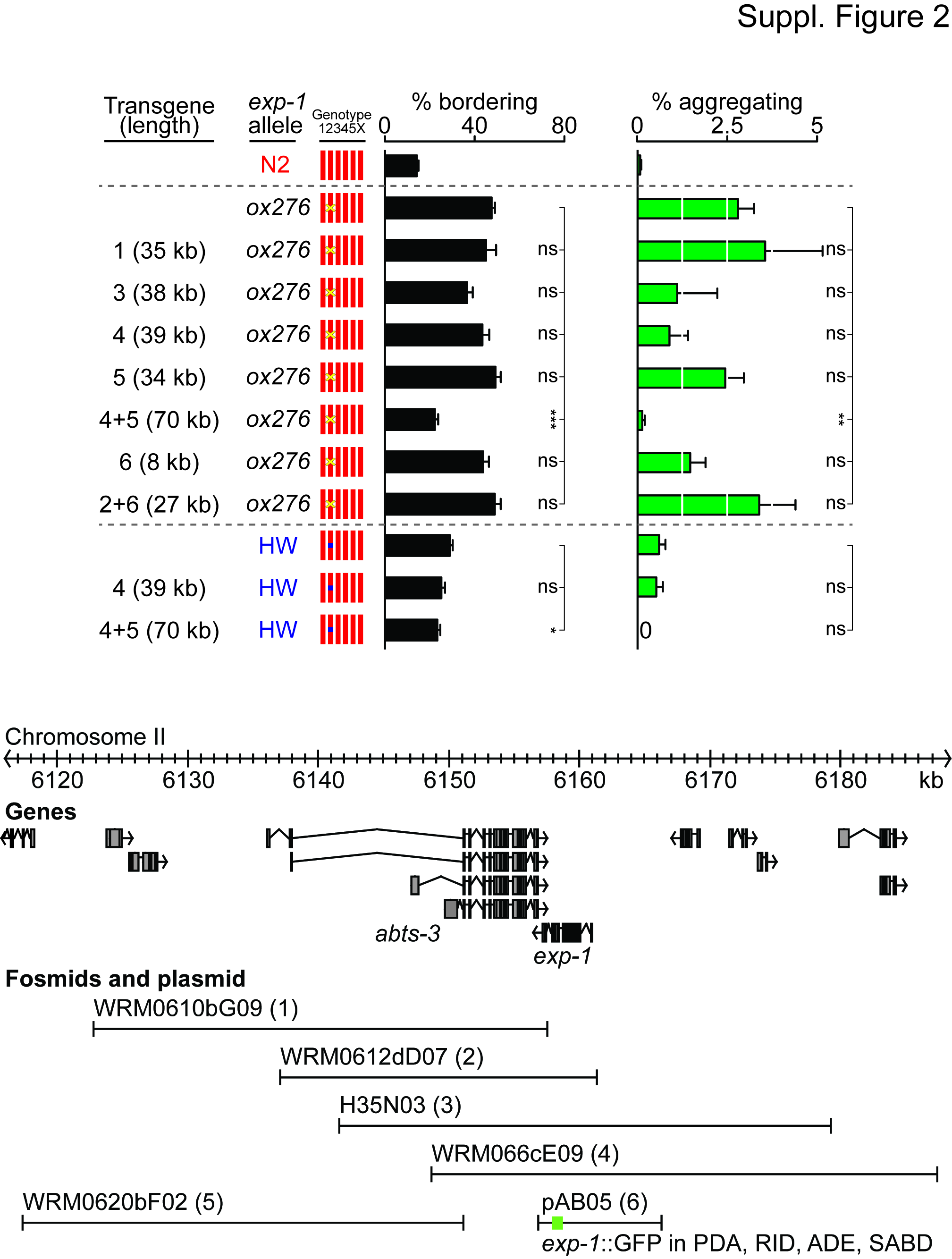

Supplement: Figure S2 — Genomic transgenes can rescue bordering and aggregation in exp-1 mutants. HW II-QTL NIL animals and exp-1(ox276) mutant animals were injected with N2-derived fosmids or plasmids depicted below. pAB05 is a genomic exp-1::GFP translational fusion that rescues the enteric defect of exp-1 but not aggregation and bordering; it is expressed in enteric muscles and in PDA, RID, ADE, and SABD neurons. Coinjection of clones (4) and (5), spanning 70 kb, rescued aggregation and bordering. At least three independent transgenic lines were tested for each injected DNA region, with consistent results. Error bars, s.e.m. * P<0.05, *** P<0.001 by ANOVA with Dunnett tests. ns, not significant. (TIF) [file pgen.1003157.s002.tif]

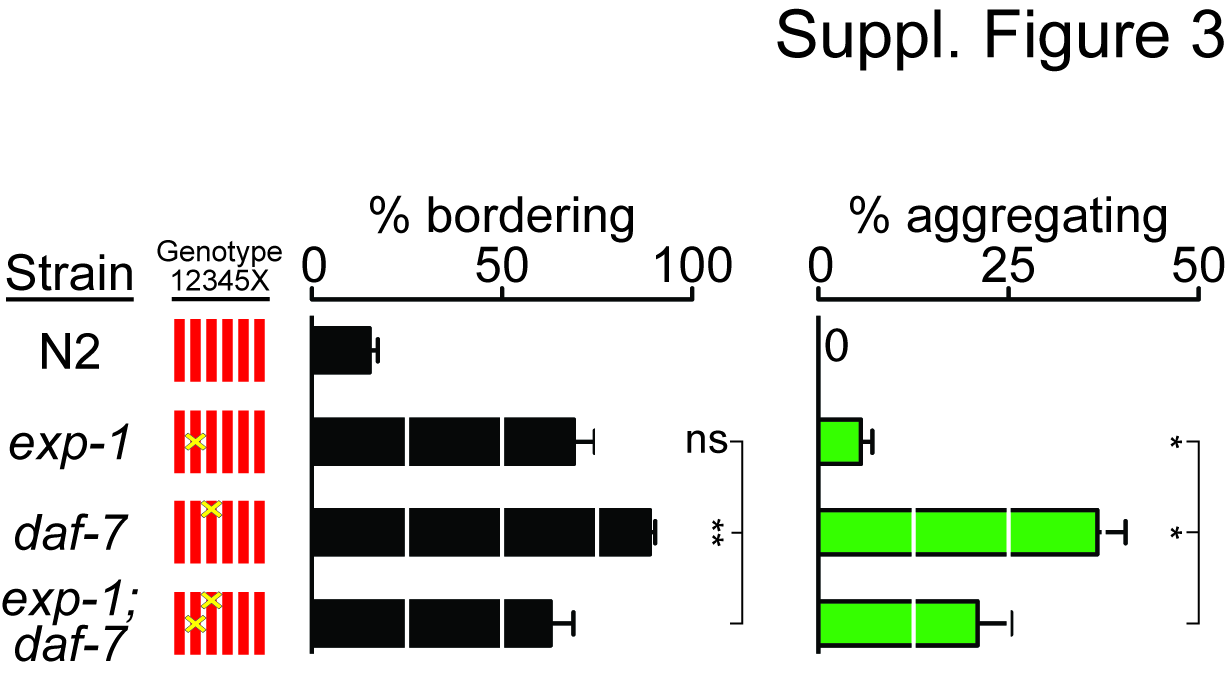

Supplement: Figure S3 — Genetic interactions between exp-1 and daf-7 in L4-stage animals. Bordering and aggregation behaviors of exp-1(ox276), daf-7(e1372), and double mutant L4-stage animals. Error bars, s.e.m. * P<0.05, ** P<0.01 by ANOVA with Dunnett test. ns, not significant. (TIF) [file pgen.1003157.s003.tif]

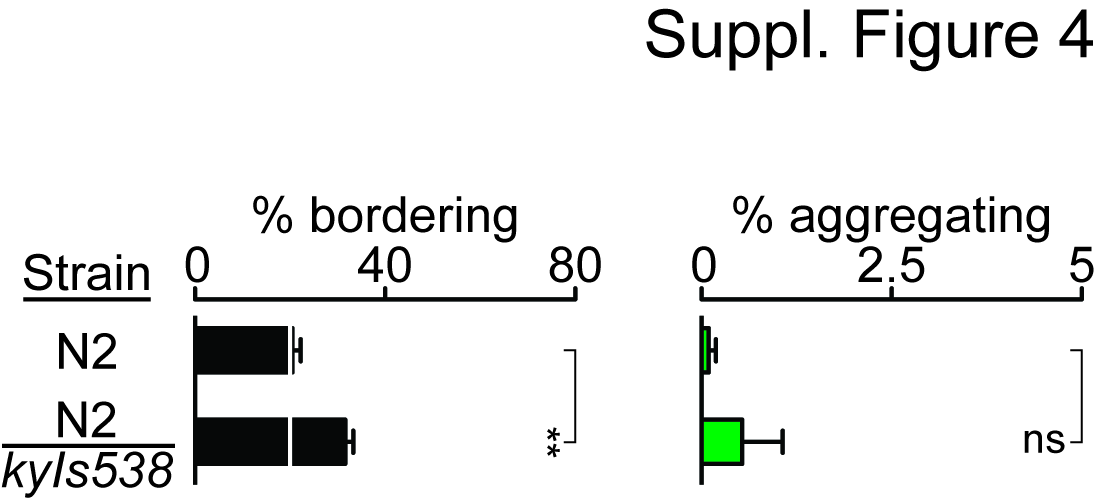

Supplement: Figure S4 — The fluorescent transgenic marker used in quantitative complementation tests has a small effect on bordering. kyIs538, an integrated mCherry marker, was used to identify F1 cross progeny in the quantitative complementation tests in Figure 3B. Error bars, s.e.m. ** P<0.01 by t-test. (TIF) [file pgen.1003157.s004.tif]

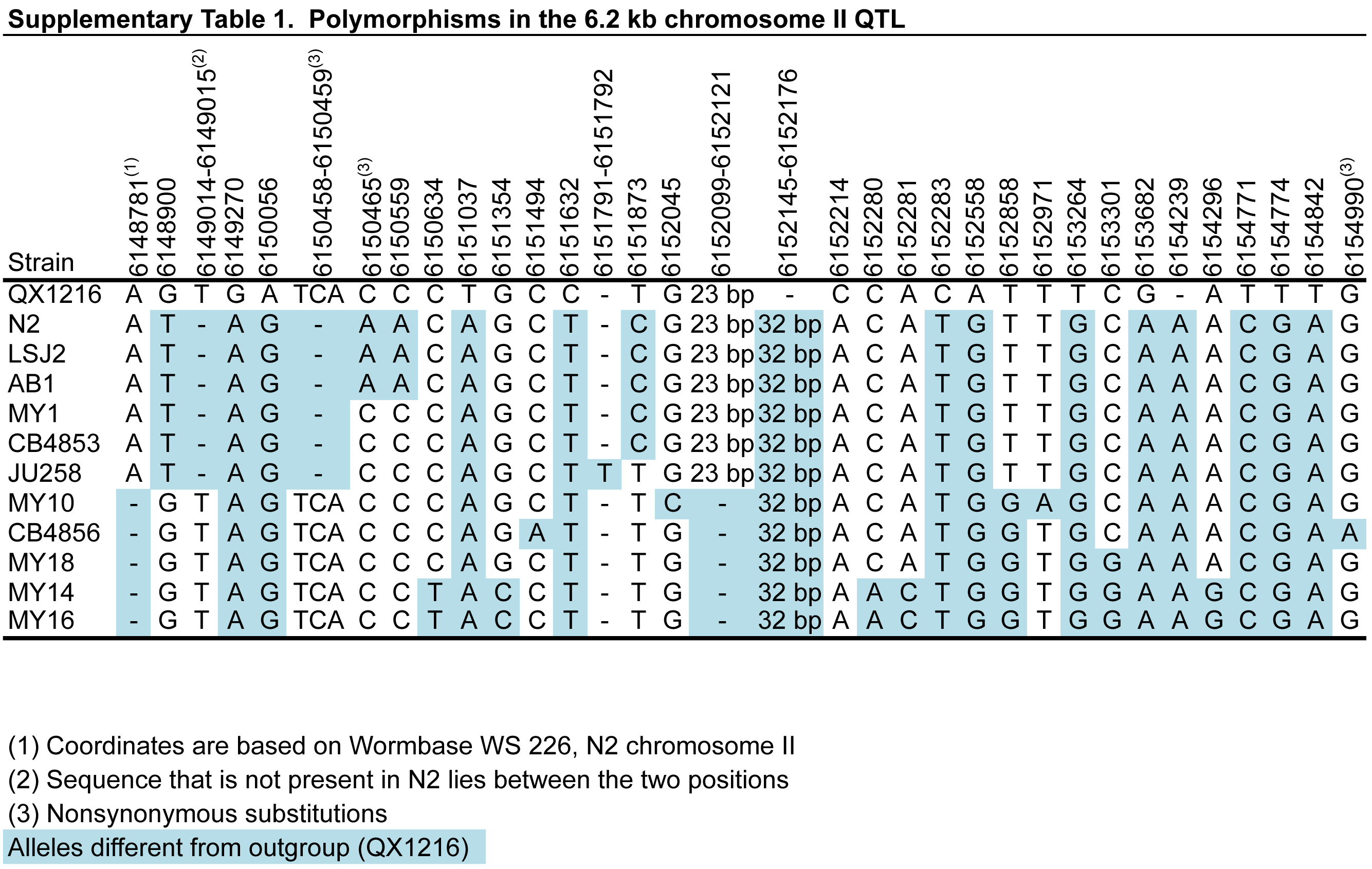

Supplement: Table S1 — Polymorphisms in the 6.2 kb chromosome II QTL. The sequence variants within the 6.2 kb QTL in 12 wild-type strains are reported relative to the sequence of the outgroup strain QX1216. (TIF) [file pgen.1003157.s005.tif]
